# Beyond pGALS: the need for a multifaceted musculoskeletal decision-making tool (‘pGALSplus’) in community-based clinical practice

**DOI:** 10.1093/rap/rkae004

**Published:** 2024-01-23

**Authors:** Vicky Mercer, Nicola Smith, Sharmila Jandial, Michela Guglieri, Simon A Jones, Helen E Foster

**Affiliations:** Translational and Clinical Research Institute, Newcastle University, Newcastle upon Tyne, UK; Children’s Physiotherapy, South Tyneside and Sunderland NHS Foundation Trust, South Shields, UK; Department of Sport, Exercise and Rehabilitation, Faculty of Health and Life Sciences, Northumbria University, Newcastle upon Tyne, UK; Translational and Clinical Research Institute, Newcastle University, Newcastle upon Tyne, UK; School of Medicine, Newcastle University, Newcastle upon Tyne, UK; Paediatric Rheumatology, Great North Children’s Hospital, Newcastle upon Tyne, UK; John Walton Muscular Dystrophy Research Centre, Newcastle University and Newcastle Hospitals NHS Foundation Trust, Newcastle upon Tyne, UK; Manchester Centre for Genomic Medicine, Saint Mary’s Hospital, Manchester, UK; Population Health Institute, Newcastle University, Newcastle upon Tyne, UK

**Keywords:** pGALS, musculoskeletal, children, assessment, triage, decision-making, allied health, rheumatology

## Abstract

Musculoskeletal (MSK) problems in children are common, and health-care professionals must identify those requiring onward referral. Paediatric gait, arms, legs and spine (pGALS) is an MSK assessment to discern abnormal joints. We aimed to identify MSK assessments to add to pGALS (pGALSplus) to facilitate decision-making in the context of exemplar conditions representing a spectrum of MSK presentations, namely JIA, mucopolysaccharidoses, muscular dystrophy and developmental co-ordination disorder. A literature review identified 35 relevant articles that focused on clinical assessments [including questionnaire(s), physical examination and functional tests] used by health-care professionals in the context of the exemplar conditions. We provide a description of these assessments and the rationale regarding how they, or components of such tools, might be useful within pGALSplus. This process provides a foundation for further work to develop and validate pGALSplus.

Key messages

- Pathways from primary/community care to specialist services are often complex, delaying access to care.
- A literature review to identify MSK assessments applicable to CYP, proposed to include in pGALSplus.
- pGALSplus assessment should include questionnaire(s), physical examination and functional tests.

## Introduction

Musculoskeletal (MSK) symptoms in children and young people (CYP) are common (one in eight in UK) [1], albeit with a wide spectrum of problems, commonly benign and self-limiting, although a minority will have serious underlying red flag conditions. Often a child with MSK symptoms is first assessed in primary or community care and by health-care professionals (HCPs) who might not be experts in MSK medicine or paediatrics. Subsequent referral for further assessment is often to hospital-based specialists (e.g. general paediatrics, rheumatology, orthopaedics or neurology/neurodisability).

The challenge in clinical practice is getting the right child to the right place and at the right time. Pathways from primary or community care to specialist services are often complex, and delay in access to care is well reported, often with an adverse effect on outcomes [2–8]. Children who are ultimately diagnosed with more serious MSK disease have often presented initially to primary care, paediatric physiotherapists or paediatric occupational therapists in the community. There is therefore a perceived need to provide further support to HCPs in the community to facilitate earlier recognition of serious MSK diseases and referral to the most appropriate specialist care.

In this article, we explore the rationale for a proposed extended paediatric gait, arms, legs and spine (pGALSplus) and we describe the comprehensive literature review completed to inform the expert interviews and focus groups to support its development. pGALSplus will be a clinical tool to facilitate assessment of CYP, probably aged between 2 and 10 years, who might have a potentially serious MSK disease. pGALSplus builds on the validated paediatric gait, arms, legs and spine (pGALS) assessment, which has been shown to discern abnormal from normal joints and is practical and acceptable in the acute paediatric setting and when performed by non-specialists [9, 10]. However, pGALS is not intended to be diagnostic but was designed to identify whether joint(s) or movement of joints are normal or not; the findings need to be interpreted in the clinical context, which will invariably entail further enquiry, physical and/or functional assessment and investigation [10–13]. With this in mind, the concept of pGALSplus aims to widen the scope of the pGALS assessment; i.e. to facilitate recognition or suspicion of serious exemplar MSK conditions, and aid decision-making for appropriate onward referral pathway(s). pGALSplus is aimed at HCPs primarily working in the community (probably physiotherapists) and those working in primary care.

Initially, a comprehensive review was undertaken to identify existing literature on MSK assessments applicable to CYP and currently used in clinical practice in the context of diagnosis and assessment within rheumatology, orthopaedics, neurodisability and neuromuscular disease. Exemplar conditions were used to enable focus, with the choice of conditions to represent a spectrum of long-term MSK pathology where clinical assessments are integral to the recognition or suspected diagnosis: developmental co-ordination disorder (DCD), JIA, muscular dystrophy (MD) and mucopolysaccharidoses (MPS). Most important is the evidence base, discussed further below, that for all chosen exemplar conditions, delay to first diagnosis and access to the right care are well reported and that early and accurate diagnosis improves long-term clinical outcomes. Furthermore, CYP with these exemplar conditions often present to community or primary care clinicians in the early stages. Clinical MSK features can be vague, such as limb pain, altered gait pattern, delay or regression of motor milestones, balance difficulties or being prone to falling. The suspicion of the diagnosis rests on clinical assessment, and confirmation usually requires specialist assessment with investigations.

Developmental co-ordination disorder is an impairment in the development of motor co-ordination that significantly interferes with academic achievement or activities of daily living [14]. Historically, there has been a lack of recognition and understanding around DCD, and delayed diagnosis limits opportunities for early intervention and timely referral [15]. DCD affects ∼5–6% of school-age children [16]. The history of DCD is of concern, not solely because of the motor co-ordination problem itself but because of its impact on everyday activities and participation [17]. The diagnosis will remain into adulthood; early intervention is essential to provide strategies and support for a CYP both at home and school, in order to maximize quality of life.

JIA is the term for a heterogeneous group of conditions affecting 1 in 1000 CYP and is the most common chronic rheumatological disease [18]. JIA is characterized by periods of disease flare that are often accompanied by pain, fatigue, morning stiffness and difficulty in performing activities both at home and at school [19]. The outcome of JIA is related to early diagnosis and to adequate referral to an appropriate specialist team [20]. Evidence demonstrates that a protracted interval between initial presentation of JIA and access to specialist paediatric rheumatology care is common [2, 21], with a study in the UK showing no significant change over a 10-year study period [3].

Duchenne muscular dystrophy (DMD) is the most common muscular dystrophy in CYP, affecting ∼1 in every 4000 male newborns [22]. Commonly reported initial symptoms include a waddling gait, difficulty with steps and falls [4]. Evidence demonstrates little reduction in the age of diagnosis in recent years [4], and in the absence of a family history it was found that there can be a delay of ∼1 year from the onset of initial symptoms to an initial appointment with a HCP [23]. Clinicians in primary care are key to suspecting DMD early, initiating specialist referral for diagnostic tests and supporting patients and their families [24]. Assessment and early diagnosis allow genetic counselling, appropriate access to standards of care, including medical treatment and physiotherapy, and allow participation in clinical research [4].

The MPS are a group of rare genetic disorders characterized by a deficiency of lysosomal enzymes [25]. The attenuated forms of MPS, atributable to their less severe presentations, are more difficult to diagnose and often receive a significant delay [26]. Undiagnosed patients with the attenuated form of MPS type I often have joint symptoms in childhood that prompt referral to a rheumatologist [27], and pGALS has been shown to detect MSK features [28]. Treatments are now available for some types of MPS, and when initiated early, can prevent damage and improve outcomes for these patients [29].

## Methods

**Figure 1.**
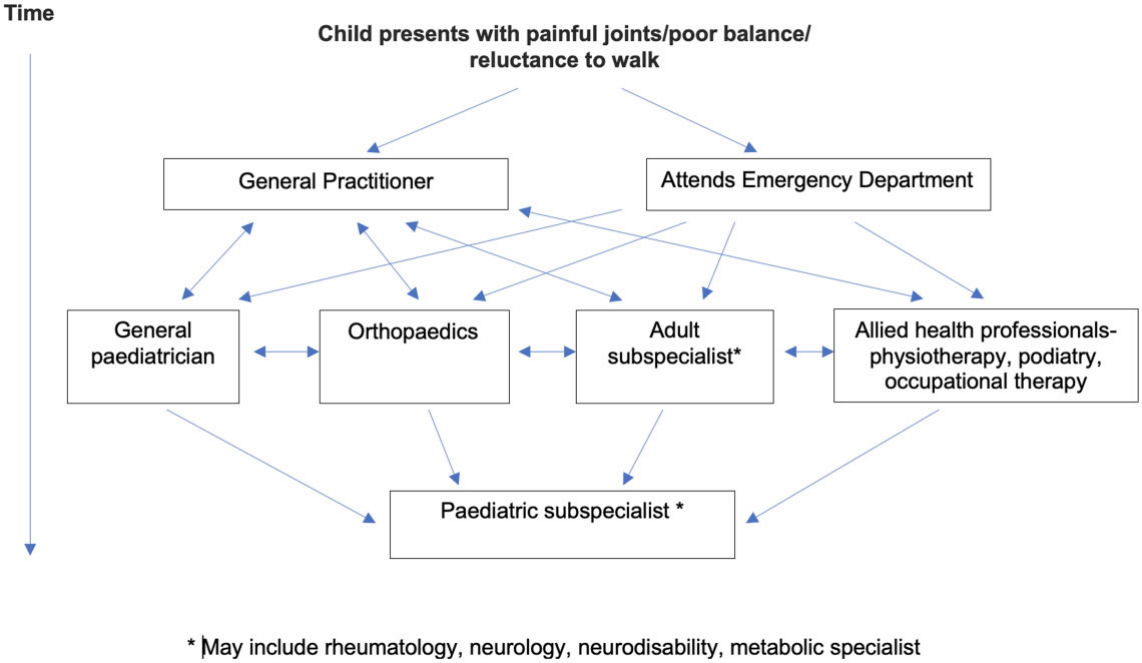
A typical journey for a musculoskeletal paediatric patient based in the UK. Figure adapted from Foster *et al.* [21]

We agreed that typical patient case scenarios, with mapped out care pathways, for each of our chosen exemplar conditions would help to identify key HCPs involved in the patient journey in order to include them as stakeholders in the development of pGALSplus. These pathways were mapped out using information from the literature and experiences of the clinical team, who provided real-world context. Fig. 1 shows an example case study scenario and highlights the complexity of current care pathways.

Scoping of the literature focused on identifying which clinical tools were available in the context of the exemplar conditions (JIA, DCD, MD and MPS) and evidence of their validity for diagnosis and assessment. The search was conducted between 1 October and 1 December 2018. The Newcastle University Library search tool was used (which includes JSTOR, Medline, Ovid, ProQuest databases, Scopus and Web of Science), in addition to Google Scholar and existing National Institute for Clinical Excellence (NICE) guidelines, pathways and clinical knowledge summaries [30]. Search terms included ‘developmental co-ordination assessment tools, dyspraxia musculoskeletal assessment, dyspraxia screening tools, paediatric balance dyspraxia assessment, paediatric musculoskeletal assessment, rheumatology screening tools children, musculoskeletal assessment in muscular dystrophy, musculoskeletal screening in muscular dystrophy, assessment of musculoskeletal abnormalities in mucopolysaccharidoses’. A snowball approach was used to identify studies cited within relevant articles uncovered through electronic searches. Article abstracts were screened initially by one researcher (V.M.) and full articles then obtained where available. Publications before 1998 were not included. Language did not constitute an exclusion criterion unless a translation into English was unavailable. Articles that referred to an adult population were also excluded. Defined variables permitted us to include only articles that described assessment or screening tools applicable to the exemplar conditions. Articles with any discrepancy around inclusion were discussed with the core research team and resolved through consensus. The authors acknowledged that an updated search was required, owing to a pause in our research activity as a result of the coronavirus disease 2019 (COVID-19) pandemic. This was undertaken in March 2023, to ensure that the information gained from the original search was still valid, and no new studies that would influence the pGALSplus assessment were identified.

### Ethical approval

Ethical approval was sought from the NHS Health Research Authority South Central-Hampshire A Ethics Committee 18/SC/0659 IRAS project: 246467. The research ethics committee was allocated as part of the Integrated Research Application System (IRAS) process.

## Results

From the original search, 35 articles were identified that described specific assessment or screening tools with an MSK component within a paediatric population. These describe 18 specific assessment or screening tools used in practice within our chosen groups (Supplementary Table S1, available at *Rheumatology Advances in Practice* online). Within the context of DCD, a number of tools exist to aid diagnosis, including the Movement ABC-2 [31], Bruininks-Oseretsky test of motor proficiency, version 2 (BOTMP-2) [32], early motor skills checklist [33] and children activity scales for parents (ChAS-P) [34]. However, the European Academy for Childhood Disability (EACD) recommendations (2012) state that motor co-ordination test batteries are not feasible as screening protocols because of both time and costs [16]. The guideline group suggest that a questionnaire might be useful as a first-step diagnostic tool, but this is not suitable for population-based screening owing to low sensitivity; the developmental co-ordination disorder questionnaire (DCD-Q) [35] is so far the best-evaluated questionnaire, although it might not be an appropriate screening tool for pre-school children owing to low test accuracy [36]. Missiuna *et al.* [17] suggest a simple parent questionnaire and clinician screening tool that can help clinicians to identify whether a child warrants further assessment. Other studies have looked at objective measures, and although these should not be used in isolation, they might be useful as part of the assessment process; for example, assessment of static balance (which has been found to be significantly worse in children with DCD when compared with a control group) [37]. Kirby *et al.* [38] reported that although there are no defined diagnostic markers for DCD, the early symptoms of motor skill difficulties can be identified during daily tasks, such as standing, walking, and throwing and catching a ball.

In the context of JIA, the early diagnosis of arthritis-12 (EDA-12) questionnaire, which can be administered by health professionals or completed by parents (and takes <5 min) [20], has been used to screen for JIA and to speed up the referral to a paediatric rheumatologist [39]. pGALS is a simple head-to-toe MSK assessment that is quick (takes ∼2 min), valid and reliable in identifying abnormal joints, in addition to being acceptable to children and families [9]. pGALS is not diagnostic of any particular MSK condition, and the findings must be interpreted in the clinical environment; pGALS was originally developed in the context of paediatric rheumatology clinics, but has been shown to detect other conditions in acute general paediatric practice, such as hypermobility, trauma, joint infection and spinal deformity [11, 12]. Other tools to assess health and wellbeing, disability and function are validated in the context of assessing disease severity and activity once the diagnosis has been made; e.g. the child health assessment questionnaire (CHAQ) [40], juvenile arthritis functional assessment scale (JAFAS) [41] and juvenile arthritis disease activity score (JADAS) [42]. A review in 2013 by McErlane *et al.* [43] described recent international developments in the assessment of disease activity and damage/disability in JIA, within both clinical practice and clinical trials.

In the context of muscular dystrophies, the DMD Care Considerations Working Group present the suspicion of a diagnosis in a flowchart, recommending that any child not walking by the age of 16–18 months and/or showing Gower’s sign (any age, but especially <5 years old) should be investigated for DMD [44]. The North Star ambulatory assessment (NSAA), developed to evaluate change in the physical abilities of ambulatory boys with DMD [45], is valid and reliable and includes skills that can be hard to complete, even in the early stages of disease, including the ability to raise their head from the floor in a supine position, standing on their heels and getting up from the floor. It is also quick to administer and freely available. The 2-min walking test appears to be able to differentiate between children with and without neuromuscular disorders, and between children with neuromuscular disorders of different ambulatory statuses [46].

With regard to MPS, the literature search did not reveal specific MSK tests pertinent to MPS, although skeletal malformations and joint problems were the presenting signs and symptoms most frequently noted by physicians, reported in >20% of cases across all MPS types [25]. It has been noted that evolving joint pain and joint contractures in the absence of inflammation should always raise the suspicion of an MPS disorder [27]. Chan *et al.* [28] described MSK abnormalities in children with MPS performing pGALS; a consistent pattern of joint involvement across various MPS subtypes was noted, and pGALS identified joints with restriction, particularly in the upper limbs, TM joints, neck, spine and hips.

### Other searches

The NICE guidelines, pathways and clinical knowledge summaries did not provide any further information around diagnosis or assessment of JIA, DCD, DMD or MPS, although there is an established clinical knowledge summary on developmental rheumatology in children that provides information around common developmental variants [47] and the use of pGALS in clinical practice.

The guideline for rheumatoid arthritis for over 16’s recognizes the importance of early diagnosis and treatment initiation [48]. A search for tools and diagnosis under a primary care perspective did not yield any pertinent results. With regard to neurodevelopment and neurodisability, a number of screening and developmental tools are available; however, the main focus is on assessing a child’s level of development.

An updated search using the same terms and inclusion/exclusion criteria was completed in March 2023. For DCD, most articles described motor co-ordination test batteries, not deemed to be appropriate for pGALSplus owing to the EACD recommendations [16], as previously discussed. These recommendations were reviewed in 2019 [49], and this recommendation remained unchanged.

Within the renewed EACD guidelines there was no longer a recommendation that a questionnaire might be useful as a first diagnostic step, but it was not suggested that this would be inappropriate. Gonzalez Lopez *et al.* [50] reported that the detection of identifiable signs (of DCD) at an early age by the family, educational and therapy professionals might imply a prior step to onward referral. We would argue that a validated questionnaire, such as the DCD-Q [35], remains appropriate as an additional resource to be included within the pGALSplus assessment, to allow HCPs to capture further information on early motor skill difficulties. Reassuringly, the review reported that signs and characteristics of functioning and contextual factors at an early age for DCD included needing more time to learn, later acquisition of dressing skills and reduced participation in recreational activities [50], all of which should be reflected in the questions asked as part of pGALSplus.

With regard to JIA, MD and MPS, the search did not identify any further clinical tools that would have influenced the development of pGALSplus. pGALS was the assessment discussed in the majority of eligible papers within the rheumatology specialty. Evidence still suggests that there is no multifaceted tool available currently that can aid decision-making by HCPs and help them to identify children with more serious presentations and thereby facilitate onward referral.

## Discussion

**Figure 2.**
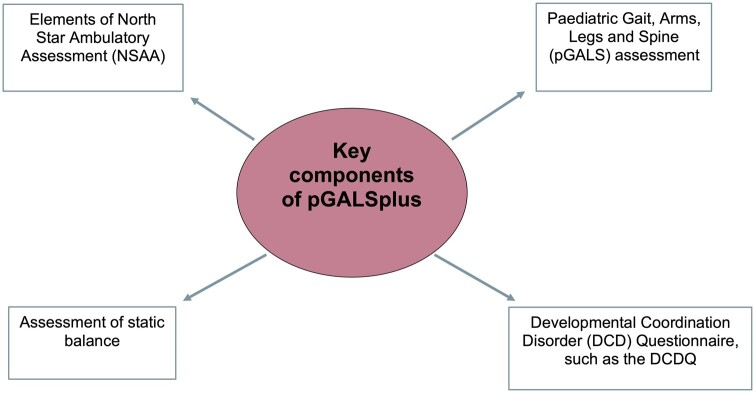
Proposed key components of the pGALSplus resource following scoping review

A pilot project completed by our research team has demonstrated that safe, effective MSK triage of CYP can be undertaken by paediatric physiotherapists working in the community [51] and involved the development and testing of triage guidance with educational resources linked to Paediatric Musculoskeletal Matters (www.pmmonline.org) [52]. The pilot project confirmed the importance of clinical assessment by allied health within the triage process in the community but did not specify what assessments are needed when suspecting different conditions. This review of the literature suggests that an MSK comprehensive assessment resource similar in type to pGALSplus does not exist currently. We propose that such a tool would be useful and enable HCPs to raise suspicion of potentially serious MSK conditions and signpost patients to appropriate specialist care as necessary. We further propose that such a resource be called pGALSplus, with inclusion of components from existing clinical resources in addition to pGALS identified from our scoping review, including an additional screening questionnaire, such as the DCD-Q [35], and assessment of static balance and components of the NSAA (Fig. 2).

### Limitations

We acknowledge a pause in research activity between the start of the initial review and the completion of the manuscript; this was attributable to the impact of the COVID-19 pandemic on the study. We also acknowledge the omission of the PRISMA-extension for scoping reviews (PRISMA-ScR) [53] within the methodology; this was not available at the time of the search, and the main focus of the literature review was to inform subsequent interviews and focus groups.

### Conclusion

The pGALSplus assessment needs to be quick and easy to be completed by younger children, and to be practical and acceptable for professionals to undertake in a busy clinical practice. It is important to engage with all stakeholders, namely allied HCPs, patients, parents and specialist teams, to ensure that the components of pGALSplus are appropriate whilst being acceptable and practical. Our next steps will focus on the iterative development of pGALSplus with stakeholder engagement and evaluating its feasability and acceptability in clinical practice. We envisage that pGALSplus wlll be an important and novel development in paediatric clinical practice to facilitate earlier recognition, prompt referral and access to specialist care and, ultimately, improve time to diagnosis and long-term clinical outcomes.

## Supplementary Material

rkae004_Supplementary_Data

## Data Availability

The data underlying this article are available in the article and its online supplementary material.
