# Supplementary material for: Beyond pGALS: the need for a multifaceted musculoskeletal decision-making tool (‘pGALSplus’) in community-based clinical practice
Source: Rheumatol Adv Pract. 2024 Jan 23;8(1):rkae004. doi: 10.1093/rap/rkae004 (PMC10822673; doi:10.1093/rap/rkae004)
Supplement: rkae004_Supplementary_Data [file rkae004_supplementary_data.docx]

**Supplementary Table S1 – Existing assessments identified within the literature for the exemplar conditions**

| **Assessment tool/**  **Questionnaire** | **Author** | **What does it assess?** | **Context of JIA, DMD, MPS, DCD** |
| --- | --- | --- | --- |
| Movement ABC - 2 | Henderson et al., 2007 [31] | Gross and Fine motor skills ‘Manual Dexterity’, ‘Aiming and Catching’, ‘Balance’ | DCD |
| Bruininks-Oseretsky Test of Motor Proficiency, Version 2 (BOTMP-2) [32] | Bruininks RH and Bruininks BD, 2005 | Fine and gross motor proficiency | DCD |
| DCD-Q-07 | Wilson et al., 2009 [35] | Screening tool for children with coordination difficulties | DCD |
| Little DCD-Q | Rihtman et al., 2012 [54] | Early identification of children with motor difficulties | DCD |
| Early Years Movement Skills Checklist | Chambers and Sugden, 2002 [33] | Movement skills in children aged 3-6 years | DCD |
| Parent questionnaire and clinician screening tool from CMAJ | Missiuna et al., 2006 [17] | Likelihood that child may have DCD | DCD |
| Children’s Self perception of Adequacy in and Pre-dilection for physical activity (CSAPPA) scale | Hay and Missiuna, 1998 [55] | Child reported screening tool for DCD | DCD |
| Children Activity Scales for Parents ChAS-P and teachers Chas-T | Rosenblum, 2006 [34] | Questionnaire designed for children aged 4-8 | DCD |
| McCarron Assessment of Neuromuscular development | McCarron, 1997 [56] | Assessment tool for DCD (fine and gross motor skills) | DCD |
| The Democritos Movement Screening Tool for preschool children (DEMOST-PRE^©^) | Kambas and Venetsanou, 2014 [57] | Screening tool for 4-6 year olds | DCD |
| Motor Observation Questionnaire for Teachers (MOQ-T). | Schoemaker et al., 2008 [58] | Screening tool for 5-11 year olds | DCD |
| Paedaitric Gait, Arms, Legs and Spine (pGALS) | Foster et al., 2006 [9] | Normal/Abnormal joints  Validated in school aged children | JIA, MD, MPS |
| EDA-12 Questionnaire | Len et al., 2006 [20] | Questionnaire to identify children with signs and symptoms of children with chronic arthropathies | JIA |
| Pediatric Pain Screening Tool (modified from the Start Back Screening Tool) | Simons et al., 2015 [59] | 9-item screening tool identifying factors associated with adverse outcomes among youth who present with pain complaints. | JIA |
| North Star Ambulatory Assessment | North Star Clinical Network for Paediatric Neuromuscular Disease (Scott et al., 2012) [45] | Functional ability (standing, walking, rising from floor) | DMD |
| 2 -min walk test | Pin and Choi, 2017 [46] | How far a child is able to walk in 2 minutes | DMD |
| Diagnostic algorithm for attenuated MPS | Cimaz et al., 2009 [27] | Algorithm to aid diagnosis | MPS |
| MPS Physical performance measure | Haley et al., 2006 [60] | Physical performance in MPS patients | MPS |

**REFERENCES**

1. Rihtman T, Wilson BN, Parush S. Development of the Little Developmental Coordination Disorder Questionnaire for preschoolers and preliminary evidence of its psychometric properties in Israel. *Research in Developmental Disabilitie 2011,s* 32(4): 1378-1387
2. Hay J and Missiuna C. Motor proficiency in children reporting low levels of participation in physical activity. *Canadian Journal of Occupational Therapy 1998, 6:64-71*
3. McCarron LT. *McCarron Assessment of Neuromuscular Development (revised edition)*. Dallas, TX: Common Market Press, 1997.

# Kambas A and Venetsanou F. The Democritos Movement Screening Tool for preschool children (DEMOST-PRE©): Development and factorial validity *Research in Developmental Disabilities 2014,* 35(7):1528-1533

1. Schoemaker MM, Flapper BCT, Reinders-Messelink HA, de Kloet A. Validity of the motor observation questionnaire for teachers as a screening instrument for children at risk for developmental coordination disorder. *Human Movement Science 2008,* 27(2): 190-199
2. Simons LE, Smith A, Ibagon C, Coakley R, Logan DE, Schechter N, Borsook D, Hill JC. Pediatric Pain Screening Tool: rapid identification of risk in youth with pain complaints. *Pain 2015,* 156(8):1511-1518
3. Haley SM, Fragala Pinkham MA, Dumas HM, Ni P, Skrinar AM, Cox GF. A physical performance measure for individuals with mucopolysaccharidosis type 1. *Developmental Medicine and Child Neurology 2006,* 48(7):576-581
